# Performance evaluation of fuzzy-logic and BP-ANN methods for WEDM of aeronautics super alloy

**DOI:** 10.1016/j.mex.2018.04.006

**Published:** 2018-04-17

**Authors:** Somvir Singh Nain, Parveen Sihag, Sunil Luthra

**Affiliations:** a Centre of Excellence in Material & Manufacturing, Department of Mechanical Engineering, CMR College of Engineering & Technology, Kandlakoya, Hyderabad-501401,Telangana, India; b Department of Civil Engineering, National Institute of Technology, Kurukshetra, 136119, India; c Department of Mechanical Engineering, State Engineering College, Nilokheri, Haryana, India

**Keywords:** Wire electric discharge machining, Fuzzy-logic, Back-propagation artificial neural network, SEM, Taguchi

## Abstract

The main purpose of this research is to check the relative importance of methods fuzzy-logic and back-propagation neural network to evaluate the performance of wire electric discharge machine (WEDM) of aeronautics super alloy. It has been confirmed that BP-ANN method reveals significant result over the fuzzy logic method for the evaluation of surface roughness and waviness of the WEDM of aeronautic super alloy. On the basis of Taguchi analysis, it has been established that the variable pulse-on, interaction amid the pulse-on and pulse-off time, wire tension and spark-gap voltage have a superlative influence on the surface roughness. The waviness is influenced prominently by pulse-on time, pulse-off time and spark-gap voltage. The thickness of recast layer is minimized up to 9.434 μm.

## Graphical abstract

**Figure:**
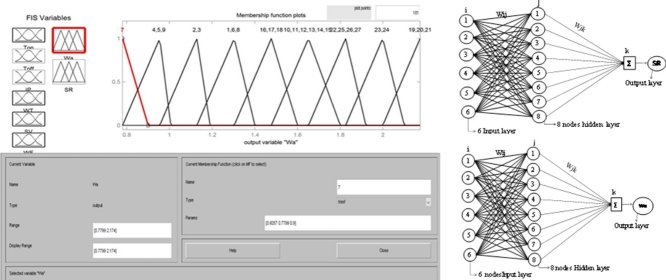

**Specifications table**

**Table:** 

*SECTION:	*Engineering*
More specific subject area:	Manufacturing
Method name:	Fuzzy-logic BP-ANN
Name and reference of original method	*Fuzzy and ANN* [18]. E.H. Mamdani and S. Assilian, An experiment in linguistic synthesis with a fuzzy logic controller, *Int. J. Man-Mach. Stud.* 7 (1), 1975, 1–13. [19]. T. Takagi and M. Sugeno, Fuzzy identification of systems and its applications to modeling and control, *IEEE Transactions of System, Man Cybern*. 15 (1), 1985, 116–132. [20]. R.J. Schalkoff, Pattern Recognition, 2003, John Wiley & Sons, Inc. [21]. C.M. Bishop, Neural Networks for Pattern Recognition, 1995, Oxford university press. [22]. S. Haykin, Neural Networks: a Comprehensive Foundation, 2nd edn., 1999, Prentice-Hall; Upper Saddle River.
Resource availability	*Mat Lab*

## Introduction

The wire-cut electric discharge machining is the machine of unique type which employed the diminutive size of 0.05 mm to 0.3 mm diameter wire for separating the infant material from the parental plate of material [1]. The sturdy electric current is allowed to flow through the wire and work material which results the generation of sturdy electrical field in the gap (0.025–0.05 mm) provided among the wire and work material [2]. Due to the high potential difference formation, a large number of distinct sparks have been generated in a close vicinity of wire and work material, therefore, the plasma zone is shaped. The material in the plasma zone is melted and evaporated. At the same instant, the enduring supply of the dielectric fluid has taken away the molted material. Eventually, the material has been extracted from the surface of the wire and work material.

Udimet-L605 is an austenitic alloy revealing the face centered cubic-crystal structure and exhibiting the non-magnetic behavior, strength and corrosion resistance at high temperature. The Udimet-L605 reveals superior resistance to air and oxidizing environment mutually [3]. It has been examined that a simple Co-base super alloy (L605) has the best impact resistance on an areal weight basis. It is 10 times better than IMI 550 (Titanium best alloy). The sterling impact resistance is delineated by Udimet L-605 primarily at velocity extra than 1100 ft/sec. For that reason, the Udimet-L605 may be the best alternative as a substitution of titanium alloy for fan containment applications in supersonic aircraft.

Hence, there is a vital need to study the surface nature after WEDM of Udimet-L605 and access the relation between the process parameters and response parameters using advanced modeling technique like back propagation artificial neural networking and fuzzy-logic. In recent years, researchers have made various efforts to evaluate the performance of WEDM on different materials using different modeling and optimization technique. The surface characteristics of WEDM generated surface has been examined [1]. The F-ANN and SA approach were applied to the WEDM process to associate the input variables with the output performances. It was noted that the cutting performance of wire-EDM can be improved using this new approach [4]. The RSM and ANN modeling of WEDM process has been recommended to pronounce the acquaintance amid the process variables and response variables. It has been evaluated that both models give accurate results for the surface roughness and material removal rates [5]. The neural network modeling of WEDM has been made to analyze the residual stress formation in electric discharge machining of metal matrix composites. It has been identified that pulse-off time have a significant effect on the residual stress formation [6]. The PCA integrated with the Taguchi approach has been recommended to identify the effect of particulate size, volume fraction, pulse-on time, pulse-off time and wire tension on SR, WWR, kerf width and white layer thickness during WEDM [7]. Taguchi approach, ANFIS modeling and grey relational analysis methods have been recommended for modeling and optimization of WEDM process [8]. The advance modeling and analysis approaches like RSM, PSO, support vector machine, regression and sensitivity analysis have been applied to investigate the performance of EDM and WEDM of advance materials [[9], [10], [11], [12], [13], [14]]. Different modeling approaches have been employed in distinct research area [[15], [16], [17]]. Based on literature study, it has been observed that a rare work has been reported on evaluation of WEDM of Udimet-L605 using fuzzy-logic and BPANN approaches.

This paper examined the change in the surface characteristics of Udimet-L605 after WEDM on Udimet-L605. The two models such as back propagation artificial neural network and fuzzy-logic have been developed to check the variance between experimental and predicted results for the surface roughness (SR) and waviness (Wa). The consistencies of the models are checked based on evaluation parameters performance of the model. Taguchi method is employed to analyze the experimental data and find the best combination of input variables for minimum surface roughness and waviness. The thickness of the white layer and a recast layer is evaluated by SEM analysis.

## Experimental details

This section consisted of three segments. The first segment demonstrates the specification, elemental composition of Udimet-L605 and its mechanical and physical properties. The second segment encompasses the details of the experimental plan and work. The third segment incorporates the measurement methodology.

### Material

A rectangular plate of Udimet-L605 has been taken as work material of dimensions 400 mm × 150 mm × 6 mm respectively. Total 81 square pieces of dimension 12 mm × 12 mm have been cut in material plate with WEDM. The chemical composition of material has been specified in Table 1.

**Table 1:** Chemical composition of Udimet-L605.

Composition	Cobalt	Chromium	Tungsten	Nickel	Iron	Manganese	Copper
Value Wt.(%)	53.2	19.0	14.4	8.44	3.20	1.20	0.46

### Experimental procedure

**Fig. 1:**
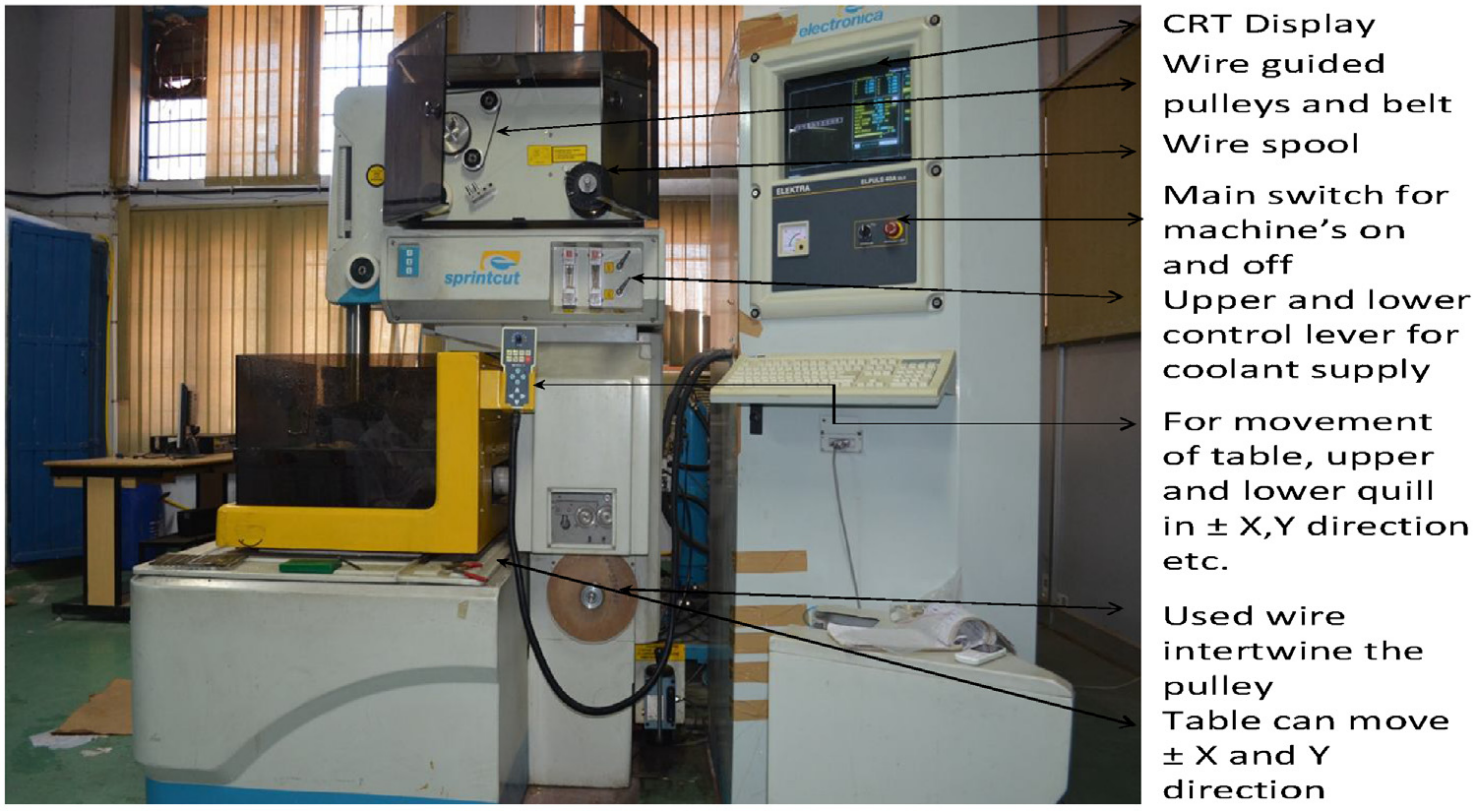
Experimental setup of WEDM machine.

**Fig. 2:**
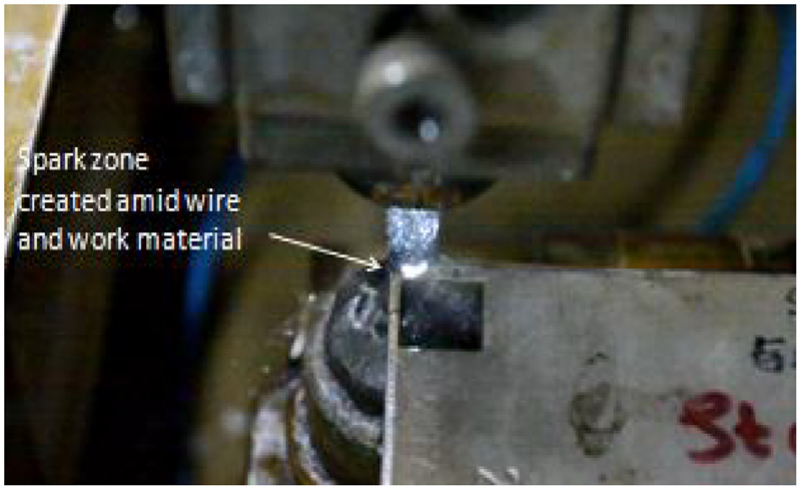
View of erosion process.

The experiments have been conducted on the Electronica sprint-cut (Electra-Elplus 40A DLX) CNC WEDM as shown in Fig. 1, Fig. 2. Plane brass wire of diameter 0.25 mm is utilized in WEDM cutting of material. Deionized water is used as a dielectric medium at constant room temperature of 25 °C.

Six input variables, namely, Pulse-on (T_on_) time, pulse-off (T_off_) time, peak current (IP), wire tension (WT), spark gap reference voltage (SV), wire feed (WF) and three one-way interactions, viz. T_on_ × T_off_, T_on_ × IP and T_off_ × IP have been chosen as input variables to elaborate the machining of Udimet-L605 with wire electric discharge machining. All of three interactions and variables were selected based on preceding literature reviews. Three levels for each input parameters have been taken as depicted in Table 2. The ranges and levels of these input parameters have been decided based on pilot experiments performed by considering five levels of each input parameter and using one factor at a time approach (OFAT).

**Table 2:** Three level input variable description.

Parameters	Designation		Level		Units
		I	II	III	
T_on_	A	106 (0.4)	114 (0.8)	122 (1.2)	Mu (μsec)
T_off_	B	28 (9)	38(13)	48 (22)	Mu (μsec)
IP	C	130	160	190	Ampere
SV	D	36	58	80	V
WT	E	1020 (7)	1260 (9)	1500 (11)	Mu (Gm)
WF	F	6	8	10	m/min

Experimentation work has been designed by means of Taguchi method of design of experimentation using a L27 orthogonal array. Based on the designed experimental layout as specified in Table 3, total 27 experiments were performed randomly and each experiment is repeated three times separately to consider the experimental error. Thus, total of 81 experiments have been performed.

**Table 3:** Experimental outcome for the L27 orthogonal array.

Run	1	2	3	4	5	6	7	8	9	10	11	12	Mean SR (μm)	SR pred. by fuzzy model	Pred.SR by BP-ANN	Mean Actual Wa (μm)	Fuzzy Model Wa	BP-ANN Wa
	A	B	A × B	A × B	C	A × C	A × C	B × C	D	E	B × C	F						
1	1	1	1	1	1	1	1	1	1	1	1	1	1.9837	1.8000	1.9930	1.3026	1.2800	1.3040
2	1	1	1	1	2	2	2	2	2	2	2	2	1.8192	1.6000	1.8220	1.0905	1.0900	1.1040
3	1	1	1	1	3	3	3	3	3	3	3	3	1.5615	1.4000	1.5640	1.0779	1.0900	1.0880
4	1	2	2	2	1	1	1	2	2	2	3	3	1.8098	1.6000	1.8110	1.0055	0.9130	1.0040
5	1	2	2	2	2	2	2	3	3	3	1	1	1.3120	1.1000	1.3130	0.9386	0.9130	0.9370
6	1	2	2	2	3	3	3	1	1	1	2	2	2.0971	1.8000	2.1060	1.2016	1.2800	1.1850
7	1	3	3	3	1	1	1	3	3	3	2	2	0.9053	1.0000	0.9100	0.7799	0.8160	0.7860
8	1	3	3	3	2	2	2	1	1	1	3	3	1.5405	1.4000	1.5490	1.1931	1.2800	1.1880
9	1	3	3	3	3	3	3	2	2	2	1	1	1.5298	1.4000	1.5360	0.9897	0.9130	0.9970
10	2	1	2	3	1	2	3	1	2	3	1	2	2.3861	2.2000	2.3940	1.5723	1.6400	1.5720
11	2	1	2	3	2	3	1	2	3	1	2	3	2.4455	2.5000	2.4470	1.7139	1.6400	1.7270
12	2	1	2	3	3	1	2	3	1	2	3	1	2.4854	2.5000	2.4870	1.7141	1.6400	1.7130
13	2	2	3	1	1	2	3	2	3	1	3	1	2.4413	2.5000	2.4460	1.6584	1.6400	1.6530
14	2	2	3	1	2	3	1	3	1	2	1	2	2.3619	2.2000	2.3640	1.6594	1.6400	1.6780
15	2	2	3	1	3	1	2	1	2	3	2	3	2.4000	2.5000	2.4090	1.5488	1.6400	1.5480
16	2	3	1	2	1	2	3	3	1	2	2	3	2.3609	2.2000	2.3710	1.5317	1.4600	1.5300
17	2	3	1	2	2	3	1	1	2	3	3	1	2.1148	2.0000	2.1190	1.4179	1.4600	1.4090
18	2	3	1	2	3	1	2	2	3	1	1	2	2.1149	2.0000	2.1230	1.5309	1.4600	1.5340
19	3	1	3	2	1	3	2	1	3	2	1	3	2.5478	2.5000	2.5490	2.1721	2.1200	2.1750
20	3	1	3	2	2	1	3	2	1	3	2	1	2.4573	2.5000	2.4640	2.1178	2.1200	2.1110
21	3	1	3	2	3	2	1	3	2	1	3	2	2.2946	2.2000	2.3000	2.1737	2.1200	2.1850
22	3	2	1	3	1	3	2	2	1	3	3	2	2.2944	2.2000	2.2970	1.8857	1.8000	1.8910
23	3	2	1	3	2	1	3	3	2	1	1	3	2.2352	2.2000	2.2410	2.1015	1.9700	2.0940
24	3	2	1	3	3	2	1	1	3	2	2	1	2.5063	2.5000	2.5070	2.0118	1.9700	2.0170
25	3	3	2	1	1	3	2	3	2	1	2	1	2.6913	2.6000	2.6910	1.8860	1.8000	1.8840
26	3	3	2	1	2	1	3	1	3	2	3	2	2.6567	2.6000	2.6640	1.9083	1.8000	1.9120
27	3	3	2	1	3	2	1	2	1	3	1	3	2.6560	2.6000	2.6630	1.9151	1.8000	1.9151

### Measurement methodology

**Fig. 3:**
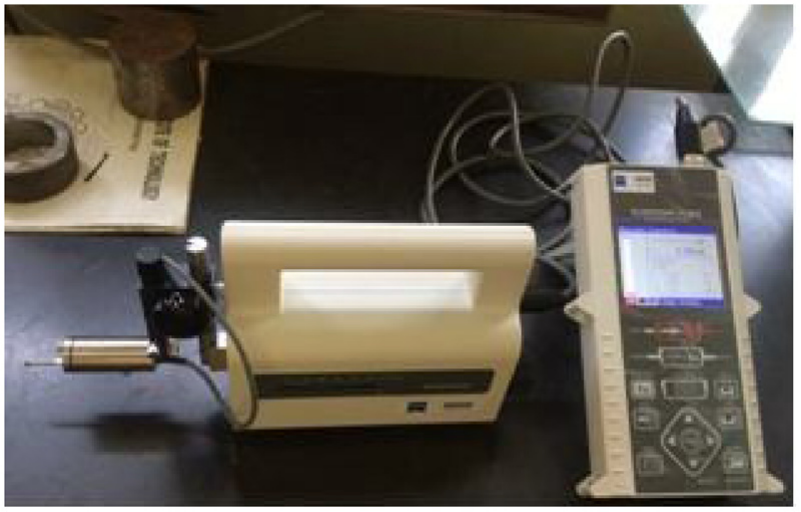
Surface roughness tester.

The surface roughness (SR) and waviness (Wa) of each experiment were measured using surfcom roughness and waviness tester as delineated in Fig. 3. The roughness and waviness of each piece have been checked on three sides of machined surfaces and three measurements were taken per surface. Thus, an average of 9 reading/sample was taken as the average roughness and waviness of each piece. The mean and predicted values of the surface roughness and waviness along with experimental layout have been given in Table 3.

## Modeling of the WEDM process

In this segment, the predicted values of surface roughness and waviness have been investigated using advanced modeling technique like fuzzy-logic and back propagation artificial neural networking. The significance of models has been identified based on graph plotted amide the actual and predicted outcome of the model for SR and Wa. In addition to this, the evaluation parameters performance like correlation coefficient (R) Nash-Sutcliffe model efficiency coefficient (NSE) and root mean square error (RMSE) was calculated to decide the prominent model. Consequently, this section consists of two segments as fuzzy-logic modeling and back propagation artificial neural network modeling for the surface roughness (SR) and waviness (Wa).

### Fuzzy-logic modeling for surface roughness and waviness

The fuzzy-logic inference engine is applied to the experimental data to identify the relation between input and output variable. Based on experimental data, the predicted value of surface roughness and waviness were attained using fuzzy inference engine. The consistency of the model is evaluated by considering three evaluation parameters like Nash-Sutcliffe model efficiency coefficient (NSE), correlation coefficient (R) and root means square error (RMSE) as depicted in Table 4.

**Table 4:** Performance evaluation result for the entire models.

	Surface Roughness				Waviness		
Model	Significant order	R	NSE	RMSE	R	NSE	RMSE
BP-ANN	1	1	0.9999	0.0057	0.9998	1	0.0079
Fuzzy-logic modeling	2	0.9787	0.9961	0.1359	0.9890	0.9981	0.0698

**Fig. 4:**
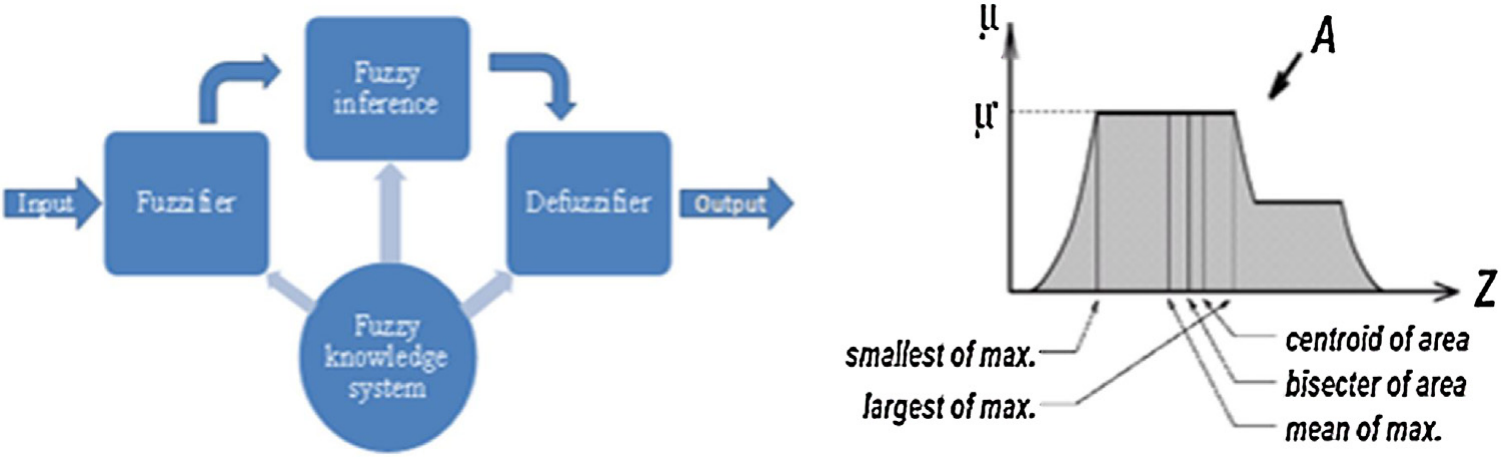
(a) Fuzzy inference engine and (b) Method of defuzzification.

Generally, a fuzzy process is a process of crisp-fuzzy-crisp for a real system. The original input and the terminal output must be crisp variables, but the intermediate process is a fuzzy inference process. The reason why one needs to change a crisp to a fuzzy variable is that, from the point of view of fuzzy control or a human being’s intuition, no absolutely crisp variable is existed in our real world. It consists of three phases as fuzzifier, fuzzy inference and defuzzifier as depicted in Fig. 4(a).

The fuzzification is the practice of mapping the crisp input and output variables into linguistics value corresponding to fuzzy memberships. It is essential to instigate the rules which are in the form of linguistic parameters. The fuzzifier takes the input data and established the limit to decide the concerned fuzzy set using membership functions. There are different types of fuzzy membership function can be used such as triangular, gaussian shape, trapezoidal and arc etc. In the present study, the triangular shape membership function is used for input and output variables as given in Eq. (1).

(1) $$\mu_{z}\left(x\right)=\left\{\begin{array}{c} 0\;\;x<p;\\ \frac{x-p}{q-p}\;\;p\leq x\leq q;\\ \frac{q-x}{r-q}\;q\leq x\leq r;\\ 0\;x>r \end{array}\;\right)$$

P and r indicates the feet of the triangular function and q represents the peak of the triangular function.

**Fig. 5:**
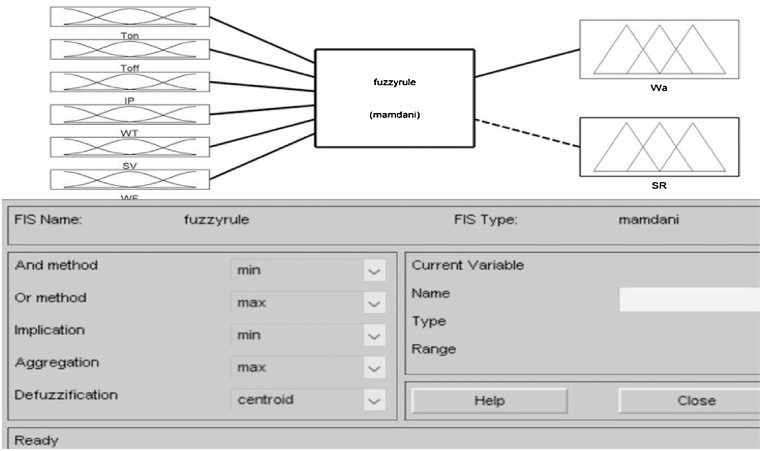
Fuzzy inference engine set communication amide the six input variables and two output variables.

**Fig. 6:**
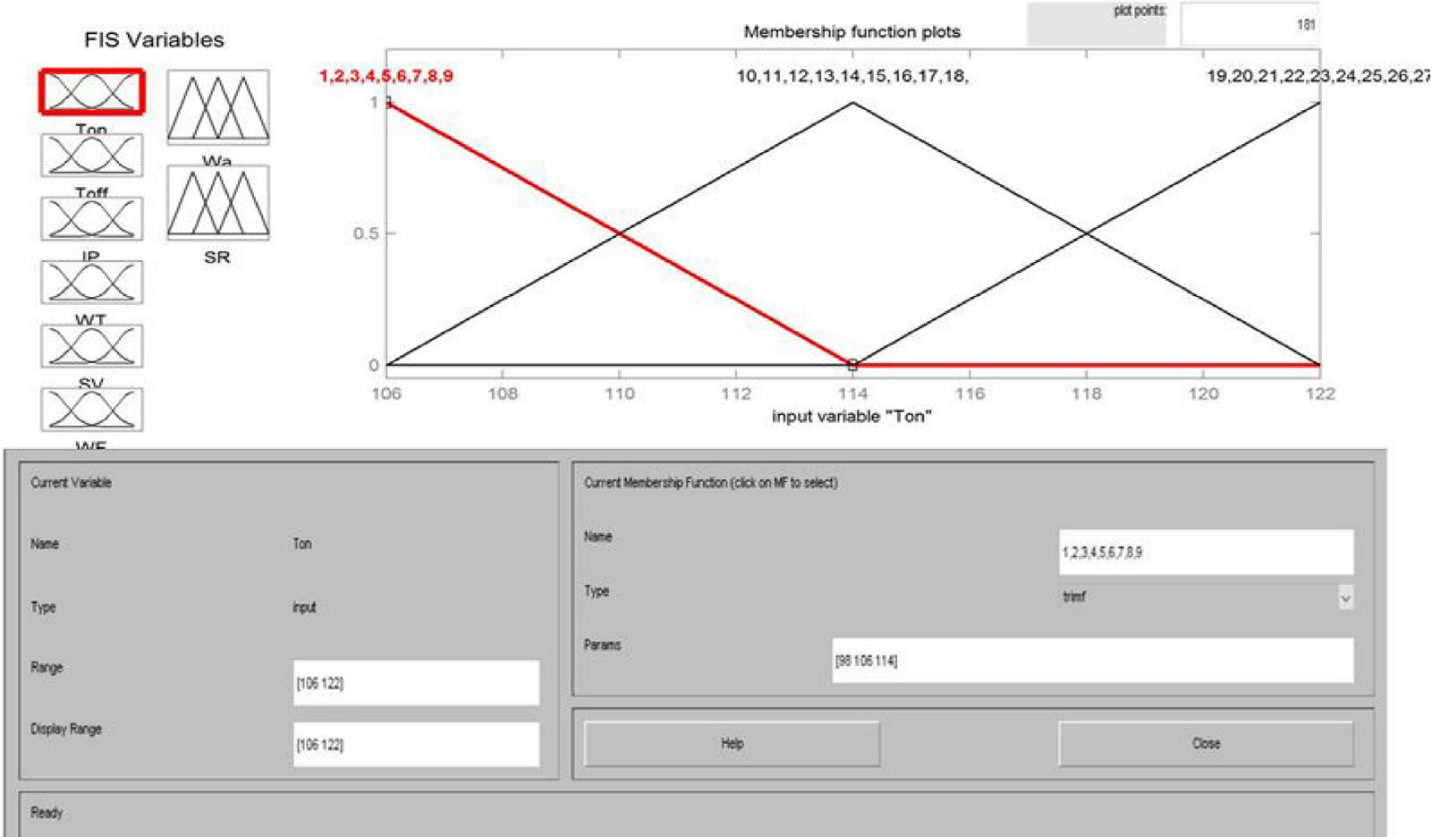
Three membership functions for input variables.

**Fig. 7:**
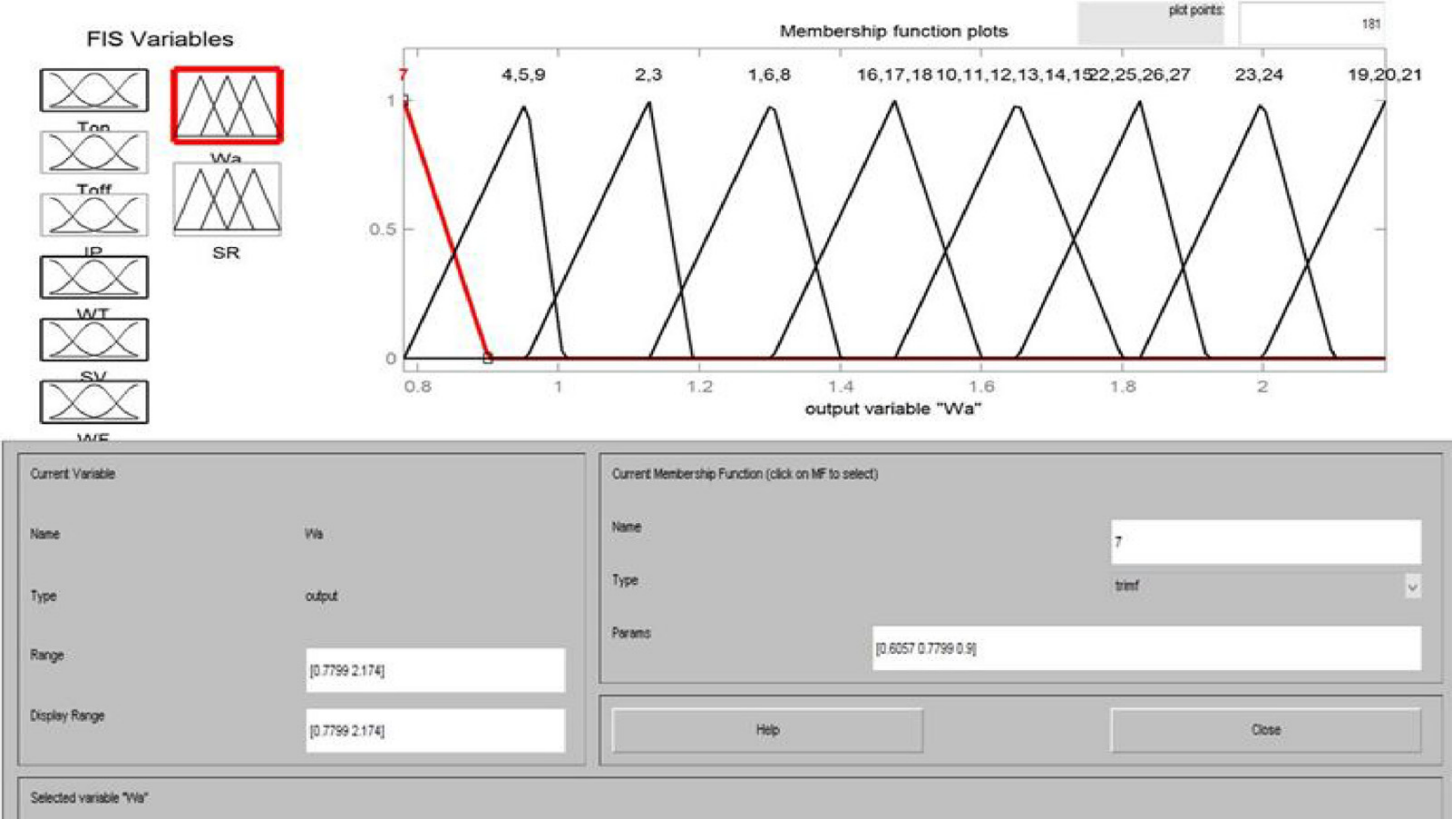
Nine membership functions for output variables.

**Fig. 8:**
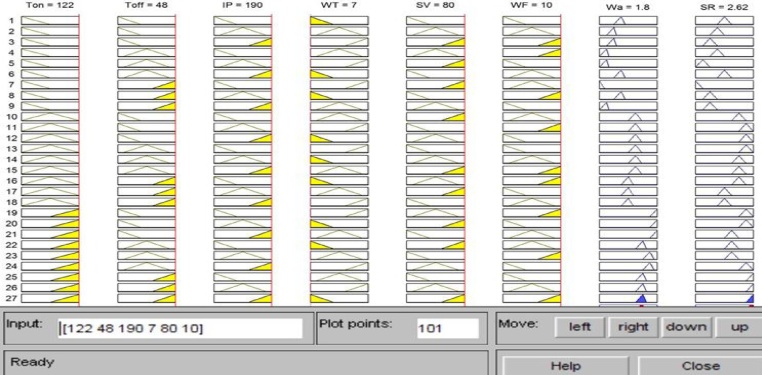
The sum of the rules considered.

Three membership functions for each process variable and nine memberships function for each response parameter were decided and communicated using the Mamdani fuzzy inference engine as depicted in Fig. 5. The fuzzy membership function for one input variables has been shown in Fig.6 and membership function diagrams for surface roughness (SR) and waviness (Wa) have been shown in Fig.7. The inputs and outputs in a fuzzy system were characterized by fuzzy rules which were decided based on experimentation and engineering expert knowledge as epitomized by Fig. 8.

Fuzzy inference process is used to combine the membership functions with the control rules to develop the fuzzy output to initiate the fuzzy inference process and arrange the fuzzy output as a lookup table. The control rules are the core of the fuzzy inference process and these rules are directly associated to a human being’s perception and feelings. In general, two most popular fuzzy inference systems are available as: Mamdani fuzzy model and Sugeno fuzzy model. The selection depends on the fuzzy reasoning and the formulation of fuzzy IF-THEN rules. Mamdani model is depended upon the collections of IF-THEN rules by means of mutually fuzzy antecedent and consequent predicts as given in Fig. 7 [18]. This model is advantageous because the rule bases were usually presented by experts. Hence, the model is lucent to clarify and study. Because of easiness, Mamdani model is still most commonly used technique for solving many real world problems.

Defuzzification process uses different methods to calculate each associated input and to characterize the output into a table: known as the lookup table. It picks up the output from the lookup table based on the current input during an application. Different methods of defuzzification can be used such as centroid method as suggest by Takagi, middle of maximum (MOM), bisector of area (BOA), last of the maximum (LOM) etc. are utilized to compute the associated control output as depicted in Fig.4. (b) and each control output should be arranged into a table called lookup table [19]. In present study centroid approach is employed for defuzzification. The centroid method (COG) is the most popular method of defuzzification and is widely utilized in actual applications as given in Eq. (2).

(2) $$Z_{COA}\;=\frac{\sum_{z}\mu_{A}\left(z\right).Z.dx}{\sum_{z}\mu_{A}\;\left(Z\right)dx}$$

Where Z_COA_ is taken as crisp output, $\mu_{A}\;\left(Z\right)$ is used as aggregated membership function and Z is taken as output variables.

### Back propagation artificial neural network

The artificial neural network (ANN) is extensively engaged for numerical prophecy and classification. It is fabricated with numbers of processing elements and comprises of three basic layers such as the input layer, hidden layer and output layer correspondingly. The channel between the layers corresponds to the weight association amid the nodes. Each processing node behaves like a biological neuron and performs mainly two functions. First, it has done the sum of the product of entire input values and weight associated with every interaction. After that, this summation is conceded over activation function f to create the outcome. By providing the weight, the network generates an outcome which is existed near to the observed target outcome as symbolized in Eq. (3).

(3) $$\;y_{j}=\Sigma W_{ij}x_{i}$$

Where *W_ij_* is symbolized as the weight considered for the channel between the unit i to j and *xi* is known as the process elements considered in the input layer. The outcome attained by employing Eq. (3) is altered by the function to generate an outcome for the j unit. The different types of activation function can be utilized, but the sigmoid function is employed in current research, which is specified as:

(4) $$f\left(y_{j}\right)=\frac{1}{1+e^{-y_{j}}}$$

The gain parameter is taken one which can change the frame of the sigmoid function by magnifying with *yj* as suggested by Schalkoff [20]. The interrelated weights were decided randomly by the network after assigning some initial weight to the network. There are a number of algorithms which can be employed to attain the negligible overall training error by adjusting the interconnected weight [21]. The generalized delta rule or back propagation is the prominent method which is generally employed for modeling the WEDM process. It is the continual process of minimizing the error between the network consequences and target consequences of the training set. The training set comprises of two data vector. The pattern is learned by a training data vector. The outcome of the training set is contained in the output vector which is achieved by the network. The main intention of the network is to minimize the error between the actual and predicted outcome of the model. This error is then fed backward through the network towards the input layer with the weights connecting the units being changed in relation to the magnitude of the error.

**Fig. 9:**
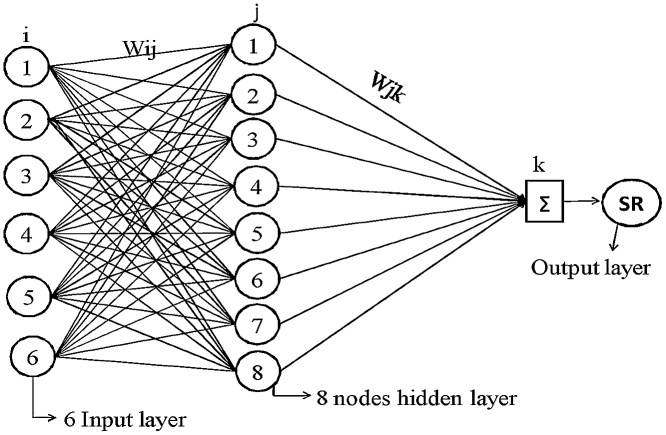
BP-ANN network for SR.

**Fig. 10:**
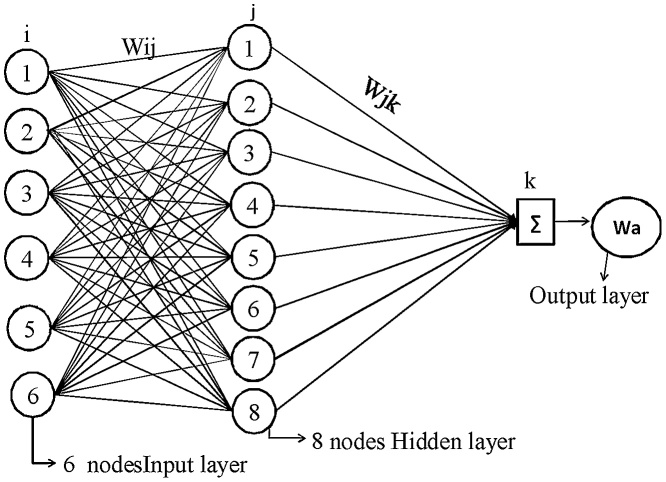
BP-ANN model for Wa.

This process is repeated until the error rate is mi nimized or reaches an acceptable level, or until a specified number of iterations have been accomplished as revealed in Fig. 9, Fig. 10. For the further details, reviewers are referred to follow the Haykin [22]. The controlling parameters of the neural networks used in the present study are the number of hidden layers, number of nodes in the hidden layers, learning rate (the amount by which the weights are updated), momentum (momentum applied to the weights during updating), and number of iterations. The value of learning rate is 0.2, momentum is 0.1 and number of iteration are 2000 with eight nodes in hidden layer respectively.

## Result and discussion

**Fig. 11:**
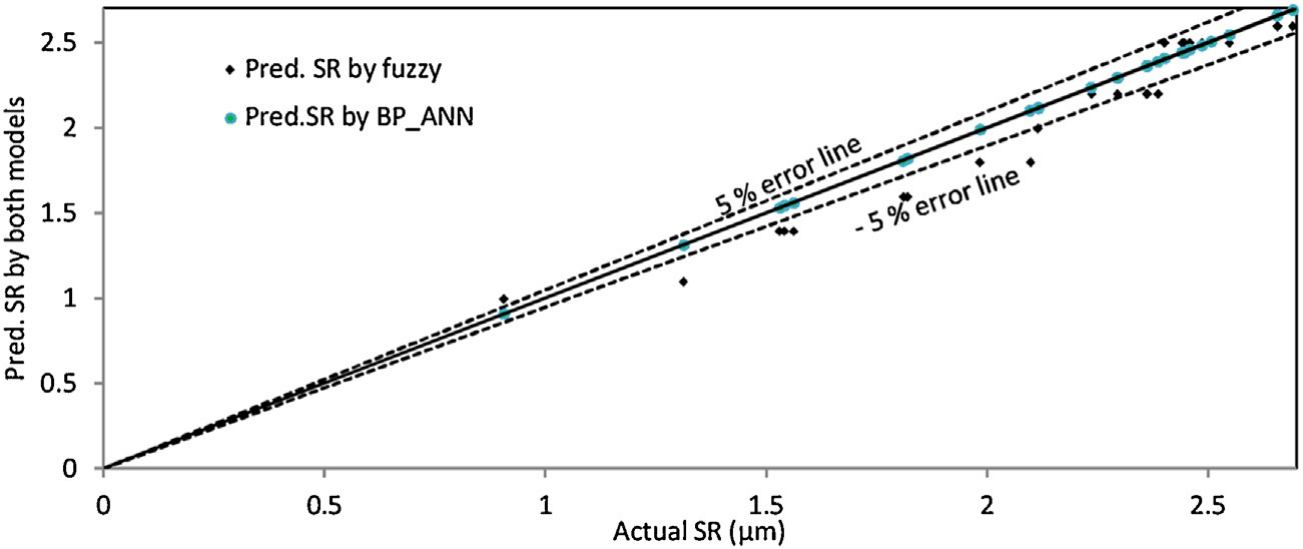
Per. error line graph between actual and predicted value of SR obtained by fuzzy and BP-ANN model.

**Fig. 12:**
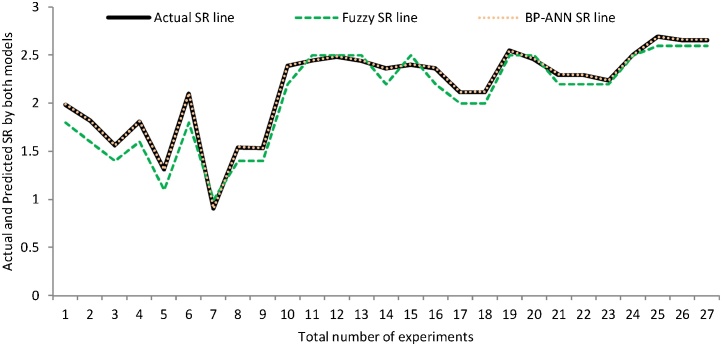
Surface roughness plot for number of experiments.

The graph as shown in Fig. 11 is plotted between the actual and predicted value of SR obtained through fuzzy logic modeling and back propagation neural network modeling. To analyze the scattering around the agreement line (i.e. line at 45 degrees) two more lines in the range of ±05% error has been plotted. It has been evaluated that most of the predicted value provided by fuzzy model and BP-ANN model lies in the range of ±05% error line which shows the consistency of both model. The BP-ANN model was dominating the fuzzy model because the most of the predicted values of SR obtained by BP-ANN model were lying on the agreement line and even the single predicted value of SR not crosses the ±05% error line as depicted in Fig. 11. While in case of fuzzy modeling, some predicted value of SR crossing the ±05% error line. Consequently, the fuzzy model was dominated by BP-ANN model. In addition to this, the graph has been plotted amid the total number of experiments and predicted value of SR by both models coupled with actual SR which prove that path followed by BP-ANN SR line travels the same path exactly as followed by actual SR line. While the fuzzy SR line (dotted line) was deviated from the actual SR line path (dark line) as depicted in Fig. 12. Therefore, it also clarifies the dominancy of the BP-ANN model over the fuzzy model. It was further confirmed by performance parameters as depicted in Table 4 which demonstrates that the value of R and NSE were obtained highest and the RMSE value was least for the BP-ANN model for SR.

**Fig. 13:**
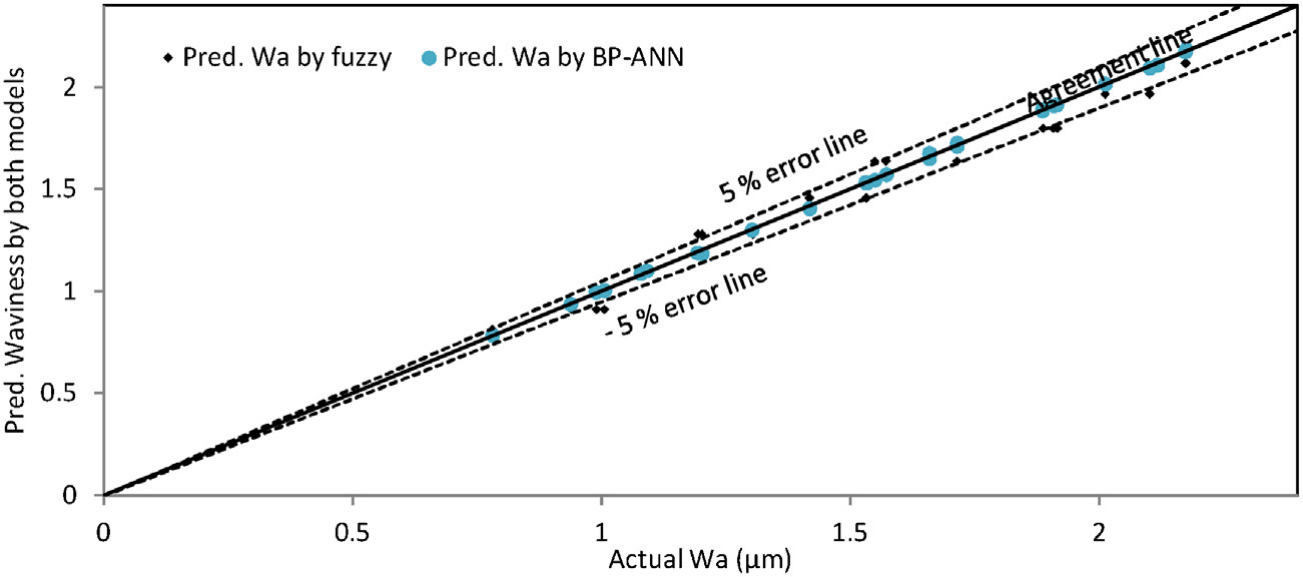
Percentage error line graph between actual and pred. val. of Wa obtained by fuzzy and BP-ANN models.

**Fig. 14:**
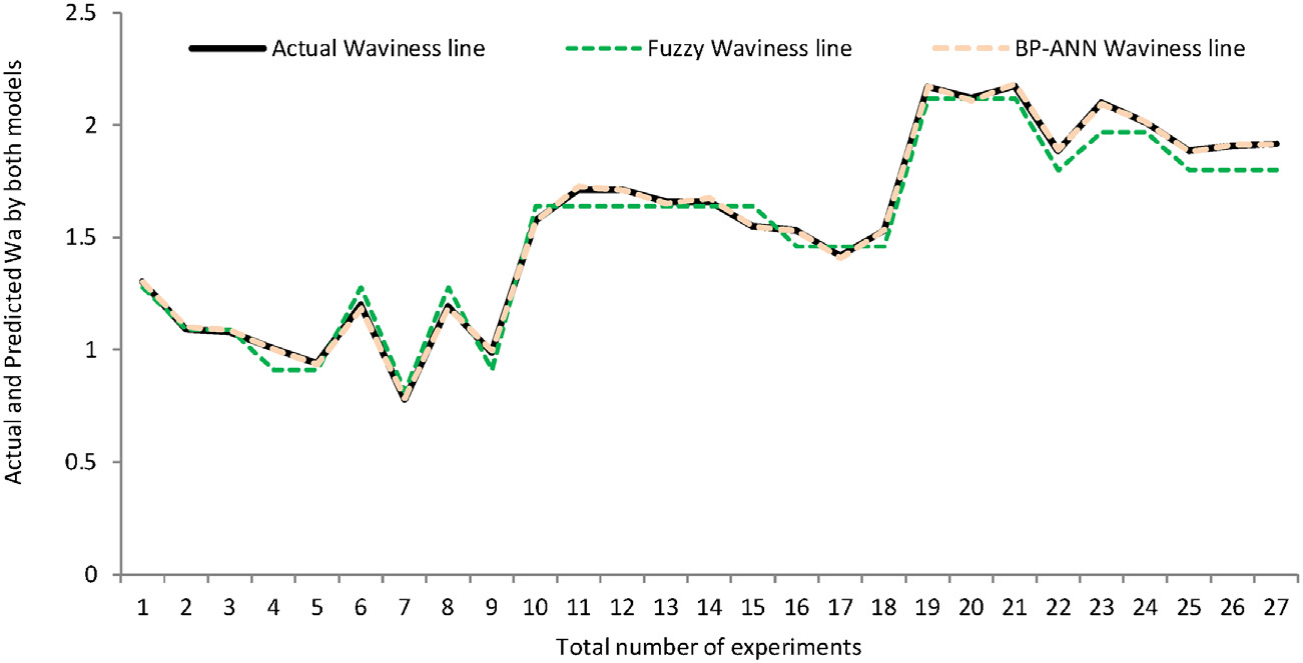
Surface waviness plot for no. of experiments.

Similarly, the graph has been plotted between the actual and predicted value of waviness provided by both models as depicted in Fig. 13. The maximum amount of predicted value of waviness existed on or around the agreement line in comparison to the values predicted by fuzzy model. Fig. 13, Fig. 14 and Table 4 demonstrated that the BP-ANN model provides the better results for waviness in contest with fuzzy logic modeling as explained for surface roughness.

**Fig. 15:**
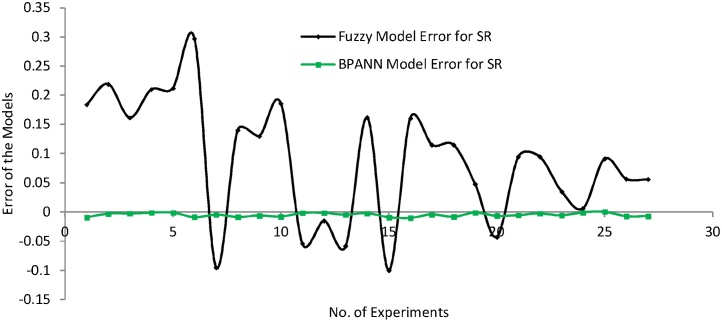
Error graph of both model for SR.

**Fig. 16:**
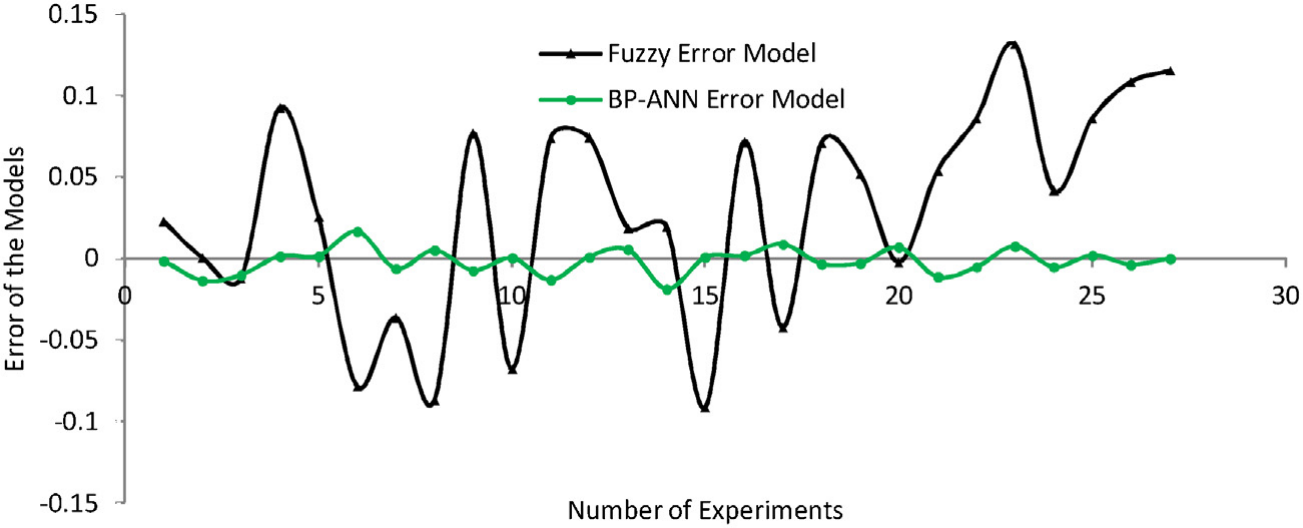
Error graph of both model for Wa.

Further the error graphs have been plotted for the predicted results of SR and Wa to validate the importance of the model relative to each other as portrayed in Fig. 15, Fig. 16. Fig. 15 demonstrates that BPANN model presents the better result in comparison to the fuzzy model for the surface roughness. Similarly, the Fig.16 also proves the dominancy of BPANN model over the fuzzy model for the waviness.

### Optimization of surface roughness and waviness

The Taguchi’s technique is used for single response optimization. The selected characteristic, surface roughness (SR) is of the type “Lower the Better”. The S/N ratio is calculated by the logarithmic transformation of loss function given by Ross (1996) [23] as shown in Eq. (5).

Surface roughness is of the type “Lower the Better”. The S/N ratio is calculated as

(5) SNratio= −10 log101n ∑i=1nyi2

ANOVA test has been conducted using the MINITAB-16 software in order to investigate the significance of input parameters. Lower the P value or higher the F value indicates the degree of importance of each input variable on surface roughness of Udimet-L605 at 95% confidence level. The insignificant parameters have been discarded from the further analysis.

The overall average of SR is delineated as: μ = 2.1485 μm

The predicted desirable outcome for the SR is delineated as:

(6) μ_SR_ =  =  (μ_A1_ +  μ_B3_ +  μ_C2_ + μ_D3_ + μ_E3_ + μ_F2_) −5 μ  = 1.088 μm

For the deliberation of confidence intervals, Eq. (7) was used as described by Ross (1996) [23].

(7) $$CI_{CE}=\;\sqrt{F_{\alpha }\left(1,\;f_{e}\right).\left\{\frac{1}{n_{eff}}+\;\frac{1}{R}\right\}.\;V_{e}}$$

Here f_e_ is exemplified as error degree of freedom = 2

F_0.05_ (1,2) = 18.513 (standardized value at 95% assurance level)

Error variance (V_e_) = 0.00074

$${\text{n}}_{\text{eff}}\;=\frac{N}{1+Tot.\;degree\;of\;freedom\;entailed\;in\;appraisal\;of\;mean}$$

N = 81, Hence, n_eff_  = 81/(1 + 12) = 6.231

R = 3 By introducing all these values in Eq. (7)

CI_CE_ = 0.006765

Thus the upper and lower bound for SR at 95% assurance level is delineated as:

μ_SR_ = 1.0813 < μ_SR_ < 1.09479.

**Fig. 17:**
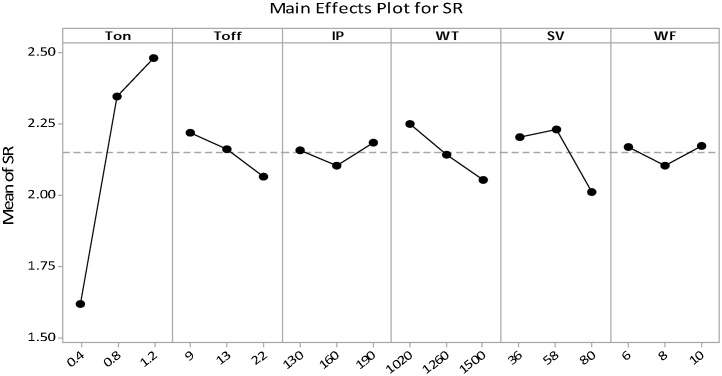
Influence of process variables on the mean value of SR.

Fig. 17 demonstrated that surface roughness increased with an increase in Ton time, IP, and WF, and decrease with an increase in wire tensions, spark gap set voltage and pulse-off time. The increment in Ton time duration integrated with decrements in Toff time duration formed the high discharge energy which results in formation of big craters and large amount of material is melted on the surface of the machined sample. The poor flushing of molten material due to the diminutive period of Toff time generated the shape of substantial layers of debris and a recast layer on the upper machined surface of the sample as entrenched by SEM image delineate in Fig. 23. The surface roughness abated with an increment in spark gap voltage due to increase in the discharge gap between the wire and workpiece which results in low discharge energy. With an increase in wire tension, the vibrations in the wire are reduced, which result in improvement in surface finishing.

**Fig. 18:**
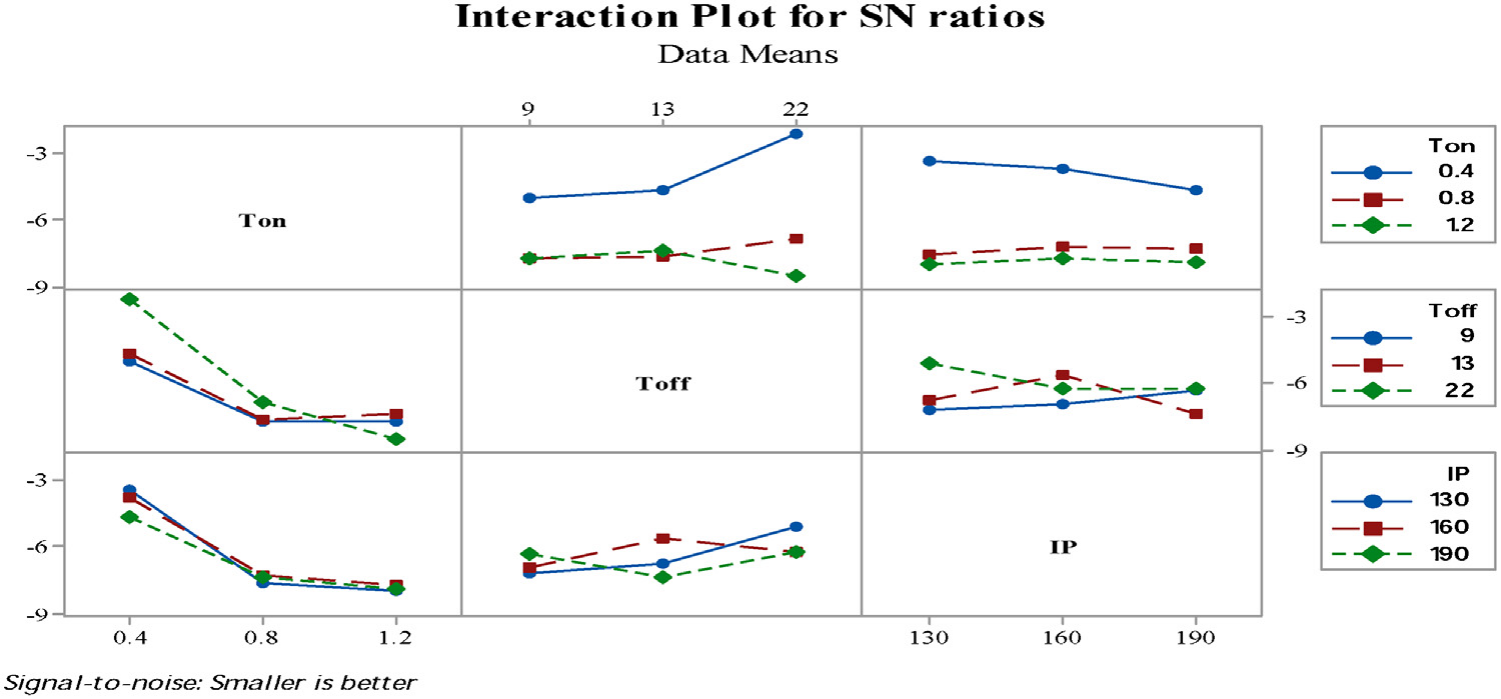
Interaction graph pertain to the S/N ratio of SR.

The Fig. 18 revealed that only interaction amid the Ton time and Toff times presents the striking persuade on the mean and the variance value of the surface roughness.

The Table 5 demonstrated that the variables Ton time, Toff time, SV, WT and interaction amide pulse-on time and pulse-off time have the significant influence on the mean and variance results for the surface roughness.

**Table 5:** ANOVA for the S/N ratio of SR.

Source	DOF	Sequ. SS	Adjo SS	Adjt. MS	F ratio	P value
A	2	82.5212	82.5212	41.2606	312.19	0.003
B	2	4.5488	4.5488	2.2744	17.21	0.055
C	2	0.714	0.714	0.3570	2.70	0.270
D	2	6.239	6.239	3.1194	23.60	0.041
E	2	8.469	8.469	4.2345	32.04	0.030
F	2	1.028	1.028	0.5139	3.89	0.205
A*B	4	13.023	13.023	3.2557	24.63	0.039
A*C	4	2.050	2.050	0.5124	3.88	0.215
B*C	4	7.876	7.876	1.9691	14.90	0.064
Residual Eroor	2	0.264	0.264	0.1322		
Total	26	126.733				

Likewise, the desirable value of waviness is described as:

The overall average of Wa is pronounced as: μ = 1.5592 μm

μ_Wa_ = = (μ_A1_ + μ_B3_ + μ_C2_ + μ_D3_ + μ_E3_ + μ_F2_) −5 μ  = 0.7864 μm and CI_CE_ = 0.03449

The upper and lower bound for the Wa at 95% assurance level is pronounced as: 0.7519 < μ_Wa_  < 0.8209.

**Fig. 19:**
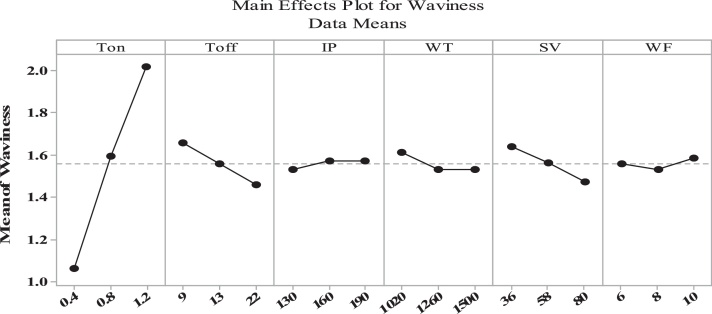
Input variables vs mean value of waviness.

The Fig. 19 asserted that waviness increased with an increase in the value of Ton time, IP and WF and reduced with an increase in Toff time, SV and WT.

**Fig. 20:**
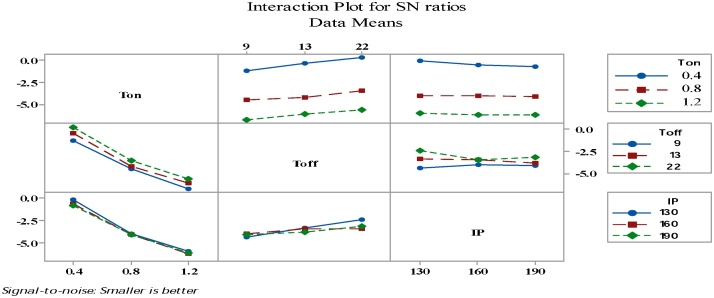
Interaction graph for the process variables vs S/N ratio of waviness.

Fig. 20 revealed that interaction didn’t have significant influence on the variance results of waviness, but the interactions between the T_on_ × T_off_ and T_off_ × IP have significant influence on mean result of Wa.

Table 6 shows that the interactions didn’t have the significant effect on variance results. The variable pulse-on time, pulse-off time, wire tension and spark-gap voltage presented the sterling effect on the mean and variance results for the Wa.

**Table 6:** ANNOVA for S/N ratio of waviness.

Source	DOF	Sequ. SS	Adjo SS	Adjt MS	F ratio	P value
A	2	146.435	146.435	73.2173	1729.72	0.001
B	2	6.128	6.128	3.0642	72.39	0.014
C	2	0.524	0.524	0.2621	6.19	0.139
D	2	2.928	2.928	1.4638	34.58	0.028
E	2	6.161	6.161	3.0805	72.78	0.014
F	2	0.559	0.559	0.2796	6.60	0.132
A*B	4	0.402	0.402	0.1006	2.38	0.317
A*C	4	0.330	0.330	0.0826	1.95	0.366
B*C	4	1.816	1.816	0.4541	10.73	0.087
Residual Eroor	2	0.085	0.085	0.0423		
Total	26	165.369				

**Fig. 21:**
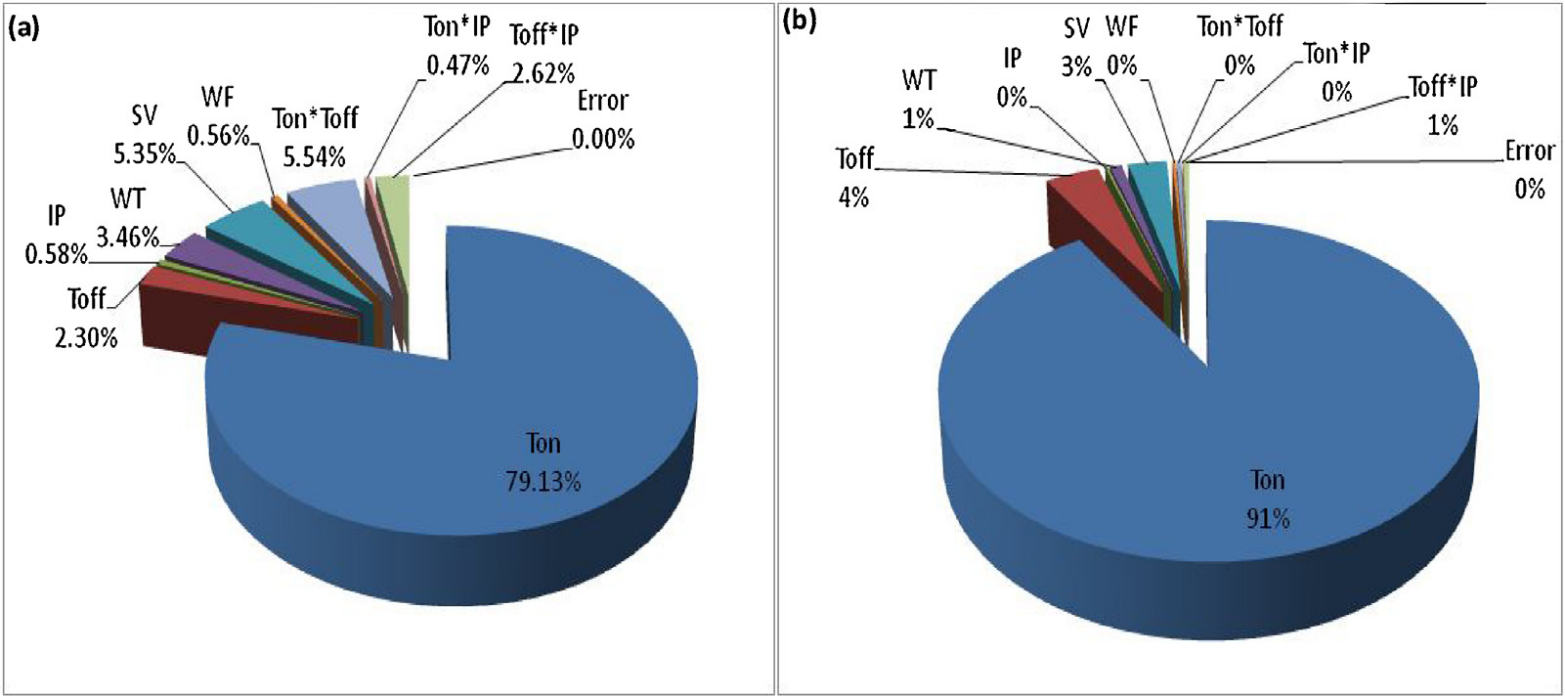
Percentage effect of variables on mean value of (a) SR (b) Wa.

Table 7 shows the optimal predicted value and confirmatory experimental value for the waviness. The confirmatory experiments were performed and repeated three times at the optimal settings of parameters. The mean value of the responses has been found to be good and existed within confidence intervals. The confirmatory experiments were reiterated thrice times at the optimal grouping of variables. The experimental confirmed value of the responses was existed within the confidence interval limit. It is confirmed that both the response variables have minimum value at the same combination of input variables as revealed by Table 7. Therefore, there is no need of employing the multi-optimization approach. The Fig. 21 illustrated the percentage persuade of input variable on mean output of SR and Wa, respectively.

**Table 7:** Optimal parameters confirmation table.

Approach	Output	Desirable setting	Predicted desirable output	Actual achived output
Taguchi optimization	SR	A1B3C2D3E3F2	1.088 μm	0.908 μm
	Wa	A1B3C2D3E3F2	0.7864 μm	0.7711 μm

## Effect of process variables on white layer and recast layer thickness

**Fig. 22:**
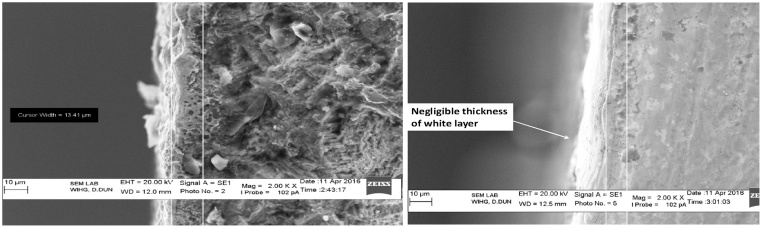
(a) White layer thickness for trial 1st (b) White layer thickness for the trial 7^th^.

The SEM analysis has been done for the trial 1th and 7th at constant lowest value of pulse-on time duration as revealed by Fig. 22(a) and (b). With an upsurge in the value of pulse-off time duration and spark gap voltage, the frequency of discharge formation has been decreased which in result, created the least amount of thermal energy. Due to minimum thermal energy generation, less amount of material is melted and evaporated from the surface of the material. Hence, the thickness of the white layer is decreased from 13.41 μm to negligible thickness as revealed in Fig. 22(a) and Fig. 22(b). Therefore, quality of the machined surface of the work material is improved during running the machine at the parameter setting for the experiment no. 7th.

**Fig. 23:**
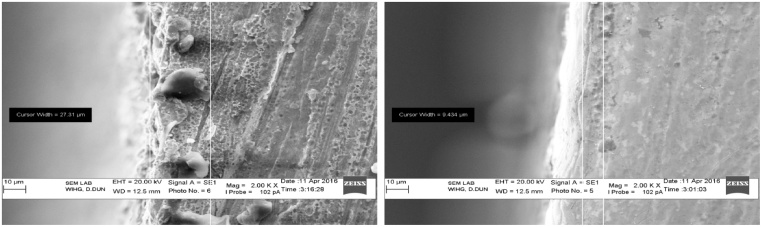
(a) Recast layer thickness for trial 1 (b) Recast layer thickness for the trial 7^th^.

In the same way, it is symbolized that the thickness of recast layer is decreased from 27.31 μm to 9.454 μm with an increase in the value of pulse-off time duration and spark-gap voltage as revealed in Fig. 23, Fig. 23(a) and (b). Hence, surface roughness and waviness were reduced with an increase in pulse-off time and spark-gap voltage. An increase in wire tension intends to decrease in vibration in the wire which also tends to decrease the surface roughness and waviness of the machined sample.

## Conclusion

(1) Both, the fuzzy and BP-ANN model presents the good result for the SR and Wa. The BP-ANN model proves its dominance over the fuzzy-logic model for both surface roughness and waviness of machined sample in WEDM of aerospace super alloy Udimet-L605.
(2) The percentage significance of input variables on the surface roughness is specified as: Ton time (79.0925%), interaction Ton x Toff (5.5373%), SV (5.3582%), WT (3.4530%), interaction Toff × IP (2.6162%), Toff time (2.300%), peak current (0.5820%), WF (0.5625%) and interaction Ton × IP (0.4681%), respectively.
(3) The percentage significance of input variables on the waviness of the machined surface is specified as: Ton time (91.15%), Toff time (03.91%), SV (2.8%), WT (0.879%), WF (0.257%), IP (0.21%), interaction Ton × Toff (0.281%), Ton × IP (0.0695%) and interaction Toff × IP (0.436%), respectively.
(4) The Ton time, interaction amid the Ton time and Toff time, SV and WT were the sterling variables for the surface roughness.
(5) The Ton ti me, SV and Toff time were the sterling variables for the waviness.
(6) The thickness of white layer was negligible at minimum thermal energy condition.
(7) The thickness of recast layer is reduced to 9.434 μm during WEDM of Udimet-L605.
